# Obituary

**Published:** 1885-06

**Authors:** J. G. Ambler


### Obituary.

### J. G. AMBLER, M.D., M.D.S.

Died suddenly of pneumonia, at his residence in Dobbs Ferry, New York, on the evening of April 6th, J. G. Ambler, M.D., M.D.S., in the sixty-ninth year of his age.

John Gardner Ambler was born in South New Berlin, Chenanago county, New York, September 2d, 1816. In the winter of 1832 he entered Rensselaer Polytechnic Institute, Troy, New York; and afterward studied medicine in Waterford, Saratoga county. He was a student of dentistry in the office of his uncle, Dr. D. C. Ambler, in New York city. He commenced the practice fohis profession in that city in 1842, and in a few years became one of the leading dentists, practicing there until the day of his death.

Dr. Ambler took an active interest in dental societies. He was one of the orginal members of the "Society of Dental Surgeons of the City of New York." He was a member of the American Dental Association, and took an especial interest in the American Dental Convention—having several times been elected president of that body—and by his energy prolonged its years of usefulness. In 1855 he edited and published the "*Dental Monitor*," a popular journal. He was probably the first dentist to issue in pamphlet form, popular instruction upon the care of the teeth under the title of "Inquiries Answered," a somewhat similar work to "The Teeth," published for the profession by the S. S. White Dental Manufacturing Co. During his professional career he contributed frequently to the literature of the profession.

In 1852 Ambler & Avery exhibited at the World's Fair, in London, the largest, the most finely finished, and the most beautiful collection of artificial dentures in metal ever manufactured, for which they received a gold medal. These specimens were also awarded a gold medal at the World's Fair, in New York, in 1853. Also a gold medal by the American Institute, New York. It was customary, and considered proper at that day, for dentists to exhibit their handiwork at the fairs.

Dr. Ambler was a communicant of the Protestant Episcopal Church, for eighteen years, being vestryman and warden of Christ Church, New York. His last literary effort was an eulogy upon the life and services of the late Rev. Geo. B. Reese, of Dobbs' Ferry, N. Y., delivered before the congregation of Zion P. E. Church of that place, on the evening of March 25th. A. T.

---

Dr. William Clendenin was born in Cumberland county, Pa., October 15, 1829. His parents were of Scotch origin, and his father was a farmer. He was very early left to the care of his mother by the death of his father. To her character and example he attributes any success or usefulness in his life. At the age of fifteen he was put in the drug store of Dr. John Gammil, at New Castle, Pa. In the doctor's family he lived, and with him studied. After four years in the store he became a regular medical student. In 1849 he attended his first course of lectures in this city, and in the spring of 1851, at the end of his second course of lectures, he graduated from the Ohio Medical College. He then spent two or three years in teaching school in Indiana. In 1853 he returned to Cincinnati, where he commenced the practice of the medical profession, in connection with the eminent Prof. R. D. Mussey, which lasted five years. In 1856 Dr. Clendenin was appointed Demonstrator of Anatomy in the Miami Medical College, with which he has been continuously connected ever since. A few years ago he was elected Dean, and as such prepared the customary "remarks" delivered for him by Prof. E. Williams at the commencement exercises of the college this spring.

In 1859 he made a trip to Europe, to further his medical knowledge. During his sojourn in Europe he studied anatomy and surgery under Velpeau, Trousseau, Malzaigue, and other eminent men of the Royal Medical College of Paris, for which he prepared himself by a study of the French language. He also attended the lectures of Sir Thomas Watson, Eramus Wilson, Sir William Ferguson, and others of the Royal College of Surgeons of London. Meantime came the outbreak of the war of the rebellion in the United States, to which country he returned, and after arriving in Cincinnati went immediately to Washington, was examined by the Medical Board, and was appointed surgeon in the army. At Camp Dennison, near Cincinnati, he had his first military experience under General Mitchel. Afterwards he was sent to West Virginia, under General Rosecrans, and there served in General Fremont's and Sigel's commands. After the second battle of Bull Run, in which he participated, he took charge of Emery General Hospital, Washington. Later he served under Rosecrans at Murfreesboro, Tenn. He became Medical Director of the Fourteenth Army Corps under General Thomas, and after the battle of Chickamauga, Assistant Medical Director of the Department of the Cumberland, with charge of all the hospitals and transfer of the sick. Subsequently he was appointed Medical Inspector of Hospitals and held the position till July, 1865. At this time he was called to Washington and received from President Johnson the appointment of Consul to St. Petersburg. But having just been chosen Professor of Surgery and Surgical Anatomy in the Miami Medical College and other private matters interfering, he was compelled to decline. It was, however, a proper recognition of Dr. Clendenin's valuable services to his country during the years of trial, which everybody now remembers, and may not be discounted by the fact that Dr. Clendenin lost his health in the war, and carried the effects of that loss through the remainder of his days to his grave. It is also part of that history that the Government not only did not grant him a pension, which he richly deserved, but failed to settle up with him for pay, simply because he was a disbursing officer, and therefore obliged to wait his turn in the circumlocution office at Washington.

Having finally returned to Cincinnati in 1865, he was appointed Health Officer, a new office created by the City Government in anticipation of the advent of cholera. He was, consequently, Cincinnati's first health officer.

After taking charge of the sanitary affairs of the city as health officer he communicated with Eastern cities in reference to their health regulations. Afterwards he drafted a sanitary bill and presented it to the legislature. This bill, with features peculiarly adapted to Cincinnati, was finally passed March, 1867. The winter before the legislature refused to pass the bill, but one year of cholera materially changed their views, and it was ultimately made a law without opposition. This did away with the council ordinance and made a permanent health organization for the city, and a basis for all the sanitary regulations of the State. To Dr. Clendenin is, therefore, due the credit of the present sanitary system of Cincinnati. He is, too, the author of the health laws of the State now in force by act of the legislature. He was one of the originators of the American Health Association and was a member of several medical associations of this city.

In both public and private life, if any distinction can be traced between them, Dr. Clendenin was a uniformly high-minded, pure man; he was the highest type of the Christian gentleman; he was a friend to the poor in the strictest sense, and hundreds of them in this city will sincerely mourn his loss. He stood in the front rank of his profession, and his memory will be honored by it and revered in its history.—*Cincinnati Commercial Gazette.*

ACTION OF THE DENTAL PROFESSION ON THE DEATH OF DR. CLENDENIN.

A called meeting of the dental profession was held at Dr. J. Taft's office, on Seventh street, May 4th, to take action on the death of Dr. Wm. Clendenin, late Professor of Descriptive and Surgical Anatomy in the Ohio Dental College of this city. Dr. J. Taft was called to the chair, and Dr. E. G. Betty was appointed Secretary. The meeting was largely attended by the leading dentists of the city, among whom were his former associates in the college faculty.

Drs. J. Taft, A. Berry, H. A. Smith, C. M. Wright, James Leslie, N. S. Hoff, and E. G. Betty were appointed committee on resolutions, which were duly reported to the meeting and unanimously adopted, as follows:

"WHEREAS, In the death of Dr. Wm. Clendenin, the dental profession of this city and of the country, through the Ohio Dental College, has lost one of its most able and successful teachers, who, during a period of nine years devoted his best efforts to the advancement of scientific dentistry by inculcating the foundation principles upon which the healing art in all its branches is based, and by his high-minded principles, his peculiar justness tempered by his kindness, his genial and courteous manners, he endeared himself to his associates in the college, to the students, and to all who knew him; therefore,

"*Resolved*, That we can not do less than place upon record our high estimation of his noble qualities as a teacher, a physician, a public benefactor, and a Christian gentleman, whom to know was to admire.

"*Resolved*, That we recognize in him a character worthy of all emulation.

"*Resolved*, That we recognize in his demise a loss to the medical and dental professions, and to the public especially, that will make a deep and lasting impression.

"*Resolved*, That our warmest sympathy is extended to his family, and that a copy of these resolutions be sent to them."

Dr. Buchanan was again found guilty by a jury at Philadelphia, last week (March 19), of issuing bogus medical diplomas. A woman named Russel, who was tried at the same time with him, was acquitted on the criminal charge. She testified that although she displayed a sign as a doctor, she had graduated from no medical school, and that M. D. following her name meant "Money Down."—*Boston Med. and Surg. Journal.*

The New York *Medical Journal* thinks that if young men had a real scientific spirit, they would ponder more over their writings before sending them to the press. They would mould and re-mould their actual material until it was in a presentable form. No doubt he is correct. But the real remedy lies in the literary education of the doctor, young or old. This is not what it should be in most cases, and hence the productions of the pens of doctors are not what they should be. Still, even these could take more care in writing without harm to themselves.

In the *Asclepiad*, Dr. Richardson calls attention to the much overlooked value of sponge as a poultice carrier, especially for mustard. After the mustard paste has been made of a smooth and even consistency it should be taken up on a clean sponge, the sponge laid in the centre of a soft, white cloth, the corners of which are tied, and the smooth convex side of the sponge is then applied to the surface of the skin. The mustard sponge, warmed again by the fire, and slightly moistened, can be applied two or three times, and remains useful for several hours. The sponge can afterwards be easily cleaned in warm water.—*Medical Press.*

